# It’s Not Always the Appendix: An Unusual Case of Right Lower Quadrant Pain in an Emergency Department Patient Due to Iliopsoas Bursitis From Uncommon Sleep Positioning

**DOI:** 10.7759/cureus.22251

**Published:** 2022-02-15

**Authors:** Susannah Boulet, Melody L Milliron, Katherine H Lund

**Affiliations:** 1 Emergency Medicine, Allegheny Health Network, St. Vincent Hospital, Erie, USA

**Keywords:** abdominal pain, right lower quadrant, sleep positioning, appendicitis, iliopsoas bursitis

## Abstract

Patients presenting to the emergency department (ED) with right lower quadrant pain will typically have acute appendicitis at the top of a limited differential. We present an unusual case of right lower quadrant pain in a 45-year-old female emergency department patient. She was referred from primary care for evaluation of suspected appendicitis with a final diagnosis of iliopsoas bursitis caused by sleep positioning. Knowledge of the appropriate evaluation and treatment of this unusual ED presentation is important for accurate diagnosis and appropriate referral to avoid unnecessary patient morbidity.

## Introduction

Iliopsoas bursitis (IB) is inflammation of the bursa dorsal to the iliopsoas muscle and is present bilaterally in 98% of adults [1]. The iliopsoas muscle is a combination of the psoas and iliacus muscles, which originate separately and join in the thigh to insert on the lesser trochanter of the femur. Together these hip flexors are key in everyday functions of walking, running, and standing. Bursae are synovial fluid-filled sacs typically located at the junction of bone and tendon where they function to reduce friction and facilitate ease of movement [1]. Most bursae are potential spaces and are not often visualized on imaging [2]. Excessive stress and inflammation can lead to enlargement of the sac and subsequent bursitis [2].

The iliopsoas bursa is the largest synovial bursa in the body [1]. Its boundaries are the iliopsoas muscle anteriorly and the hip posteriorly. The femoral artery and femoral vein border medially [2]. This bursa extends from the inguinal ligament to the lesser trochanter of the femur. Diagnosing iliopsoas bursitis on history and physical exam alone can often be difficult, and imaging is often required.

## Case presentation

**Figure 1:**
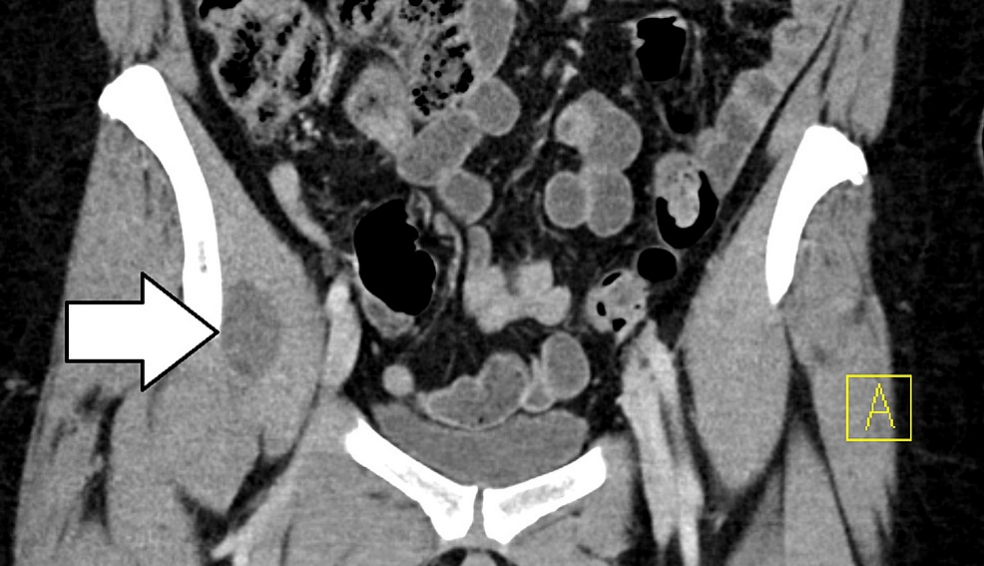
CT with contrast (coronal image) with right-sided iliopsoas bursitis. CT: computed tomography.

**Figure 2:**
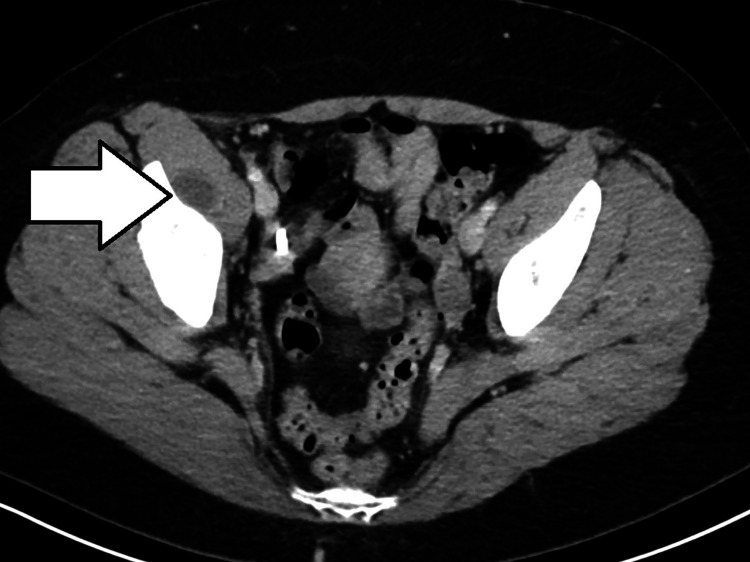
CT with contrast (transverse image) with right-sided iliopsoas bursitis. CT: computed tomography.

A 45-year-old female presented to the emergency department (ED) with a chief concern of right lower quadrant abdominal pain. Her pain began gradually over several months and became more noticeable over the preceding three weeks. She characterized the pain as aching and bloating with no exacerbating or alleviating factors. She was assessed by her primary care provider, who referred her to the emergency department for evaluation of possible appendicitis. She denied any associated symptoms such as fevers, chills, anorexia, painful urination or urinary frequency, vaginal bleeding, or discharge. She denied any prior history of nephrolithiasis or ovarian cysts. A physical exam revealed a well-appearing 45-year-old female in no acute distress. Initial vital signs were pertinent for tachycardia at a rate of 108 beats per minute. The patient was afebrile, and her blood pressure, respiratory rate, and pulse oxygenation were within normal limits. Her abdominal exam showed a soft, non-distended abdomen with normal bowel sounds, no rigidity, rebound, or guarding. The right lower quadrant was tender to deep palpation and pain was elicited with internal and external rotation of the right hip. Laboratory evaluation included a complete blood count with differential, a comprehensive metabolic panel, lactic acid, lipase, pregnancy testing, and urinalysis. All were normal. Inflammatory markers were not obtained. A computerized tomography (CT) scan of the abdomen and pelvis with intravenous (IV) contrast was obtained (Figures 1, 2).

CT showed fluid distension of the right iliopsoas bursa suggesting iliopsoas bursitis without other acute abnormality. Results were reviewed and discussed with the on-call orthopedic physician. Upon discussion with the patient, she recalled frequently sleeping with her right leg tucked under her for many years. She was subsequently counseled on sleep positioning precautions. Due to the patient’s history of Roux-en-Y surgery for treatment of obesity, non-steroidal anti-inflammatory drugs (NSAIDs) and prednisone were thought to have a higher risk of significant side effects, and the patient was discharged with cyclobenzaprine. She was given follow-up instructions with sports medicine and orthopedic surgery as well as ED return precautions.

## Discussion

Iliopsoas bursitis is an infrequent cause of hip or abdominal pain and is not a well-known cause of right lower quadrant pain. A limited differential of right lower quadrant pain includes appendicitis, ovarian cyst, ovarian torsion, pregnancy, ureterolithiasis, diverticulitis, colitis, pyelonephritis, cystitis, and pelvic inflammatory disease. Additional history and physical exams can narrow this differential. However, a musculoskeletal or hip etiology is not often included in the initial differential. Providers should recognize that symptoms may arise from irritation or compression of local structures. Patients with iliopsoas bursitis typically complain of anterior hip pain that is exacerbated by motions that flex the hip, including walking, standing, and climbing stairs [3]. Diagnosis on the clinical exam alone is difficult, and typically imaging is necessary [4]. Superficial bursitis can be seen on ultrasound imaging, whereas a deeper bursa requires magnetic resonance imaging (MRI) or computed tomography (CT) [2,4]. As with our case, iliopsoas bursitis is seen as a hypodense collection with an enhancing wall on CT. It is important to distinguish iliopsoas bursitis from similar appearing differentials, including hemorrhage, abscess, aneurysm or fistula, lymphadenopathy, hernia, or neoplasm [2]. Iliopsoas bursitis is frequently caused by overuse injuries resulting from excessive use or strain of local structures. Other causes of bursa enlargement may be attributed to infection, trauma, arthropathies, avascular necrosis, and synovial chondromatosis [2]. Due to their proximity, iliopsoas bursitis and tendinitis of the iliopsoas tendon are frequently interrelated [1]. When combined, this condition is termed "iliopsoas syndrome" [1].

Treatment options for over-use injury bursitis are typically conservative and include over-the-counter agents NSAIDs, including ibuprofen and naproxen. For patients at higher risk of NSAID-induced side effects, acetaminophen may also be an option for pain control. Patients should be referred to physical therapy, sports medicine, or orthopedic surgery for guidance on activity modification, stretching, posturing, and hip flexor strengthening. On an outpatient basis, localized corticosteroid injections can be considered for refractory pain and inflammation. Surgery is not usually necessary [5].

## Conclusions

Iliopsoas bursitis is an atypical diagnosis in an ED patient presenting with right lower quadrant pain. The diagnosis can be challenging and does not often appear on the initial differential list. The differential should include various causes of right lower quadrant pain, with additional consideration for a musculoskeletal etiology. Diagnosis can be difficult to obtain solely on history and physical exam alone, but typical features include pain exacerbated with hip flexion. Patients may not report these symptoms on initial history acquisition. Imaging is often crucial for a definitive diagnosis. For most patients, conservative treatment is indicated with NSAIDs or acetaminophen, and appropriate outpatient referral. As a patient presenting with right lower quadrant pain has concern for acute surgical etiologies such as acute appendicitis, an aggressive search for the underlying diagnosis is critical. Although an uncommon cause of right lower quadrant pain, iliopsoas bursitis is an important addition to the differential of right lower quadrant pain to allow early ED diagnosis and thereby minimize patient morbidity.
